# Remote Sensing Energy Balance Model for the Assessment of Crop Evapotranspiration and Water Status in an Almond Rootstock Collection

**DOI:** 10.3389/fpls.2021.608967

**Published:** 2021-03-10

**Authors:** Joaquim Bellvert, Héctor Nieto, Ana Pelechá, Christian Jofre-Čekalović, Lourdes Zazurca, Xavier Miarnau

**Affiliations:** ^1^Efficient Use of Water in Agriculture Program, Institute of Agrifood Research and Technology, Fruitcentre, Parc Científic i Tecnològic Agroalimentari de Lleida, Lleida, Spain; ^2^Complutum Tecnologías de la Información Geográfica, Madrid, Spain; ^3^Fruit Production Program, Institute of Agrifood Research and Technology, Fruitcentre, Parc Científic i Tecnològic Agroalimentari de Lleida, Lleida, Spain

**Keywords:** thermal, field phenotyping, water productivity, TSEB model, stem water potential, crown area, yield

## Abstract

One of the objectives of many studies conducted by breeding programs is to characterize and select rootstocks well-adapted to drought conditions. In recent years, field high-throughput phenotyping methods have been developed to characterize plant traits and to identify the most water use efficient varieties and rootstocks. However, none of these studies have been able to quantify the behavior of crop evapotranspiration in almond rootstocks under different water regimes. In this study, remote sensing phenotyping methods were used to assess the evapotranspiration of almond cv. “Marinada” grafted onto a rootstock collection. In particular, the two-source energy balance and Shuttleworth and Wallace models were used to, respectively, estimate the actual and potential evapotranspiration of almonds grafted onto 10 rootstock under three different irrigation treatments. For this purpose, three flights were conducted during the 2018 and 2019 growing seasons with an aircraft equipped with a thermal and multispectral camera. Stem water potential (Ψ_*stem*_) was also measured concomitant to image acquisition. Biophysical traits of the vegetation were firstly assessed through photogrammetry techniques, spectral vegetation indices and the radiative transfer model PROSAIL. The estimates of canopy height, leaf area index and daily fraction of intercepted radiation had root mean square errors of 0.57 m, 0.24 m m^–1^ and 0.07%, respectively. Findings of this study showed significant differences between rootstocks in all of the evaluated parameters. Cadaman^®^ and Garnem^®^ had the highest canopy vigor traits, evapotranspiration, Ψ_*stem*_ and kernel yield. In contrast, Rootpac^®^ 20 and Rootpac^®^ R had the lowest values of the same parameters, suggesting that this was due to an incompatibility between plum-almond species or to a lower water absorption capability of the rooting system. Among the rootstocks with medium canopy vigor, Adesoto and IRTA 1 had a lower evapotranspiration than Rootpac^®^ 40 and Ishtara^®^. Water productivity (WP) (kg kernel/mm water evapotranspired) tended to decrease with Ψ_*stem*_, mainly in 2018. Cadaman^®^ and Garnem^®^ had the highest WP, followed by INRA GF-677, IRTA 1, IRTA 2, and Rootpac^®^ 40. Despite the low Ψ_*stem*_ of Rootpac^®^ R, the WP of this rootstock was also high.

## Introduction

The study of the behavior of *Prunus* cultivars grafted on different rootstocks in fruit production serves to adapt cultivars to different edaphic and environmental conditions and to enhance sustainable crop production. The selection of a suitable scion-rootstock combination is the first step to monitor the vegetative growth, yield and fruit composition parameters of the scion (Caruso et al., 1996; Mestre et al., 2017; Font i Forcada et al., 2020; Reig et al., 2020). There is growing interest in selecting and breeding new rootstocks and cultivars with a higher water use efficiency (WUE) in order to improve water productivity and better adapt fruit production to future climate changes (Solari et al., 2006; Xiloyannis et al., 2007; Díez-Palet et al., 2019). In almonds [*Prunus dulcis* (Mill.) DA Webb], with the recent introduction of high-density planting systems, particular attention has been paid to using dwarf rootstocks in order to control canopy vigor and facilitate mechanical harvesting (Pinochet, 2009; Casanova-Gascón et al., 2019). In addition, with the introduction of new dwarfing rootstocks and hybrids coming mainly from the peach sector, the paradigm has changed since information about their response to drought or a limited water supply is scant.

For many years, breeding programs for fruit crop rootstocks, as well as for obtaining scion cultivars, have used similar evaluation methods based on both agronomic and molecular traits. Some of the commonly measured agronomic traits are trunk cross-sectional area (TCSA), plant height, tree canopy vigor, phenology, yield parameters, and fruit quality attributes (Reighard et al., 2011; Font i Forcada et al., 2012; Legua et al., 2012; Lordan et al., 2019). However, most of these agronomic traits are a consequence of differences in the root system architecture or the hydraulic properties of a rootstock, which contribute in influencing the transpiration rate through their effects on the stem water potential (Ψ_*stem*_) and the control of stomatal conductance (Hernandez-Santana et al., 2016). On the other hand, the development of markers to help select individuals with traits that are complex to evaluate should speed up the development of new rootstocks that are resistant or tolerant to multiple biotic or abiotic stresses (Cantini et al., 2001; Arismendi et al., 2012; Jiménez et al., 2013; Guajardo et al., 2015). However, the types of methodology required for this remain fairly time-consuming, costly and, in some cases, are still scarce.

In recent years, proximal and remote sensing (RS) technologies have increasingly been used to assess vegetation in the context of field-based phenotyping (FBP) (Deery et al., 2014; Araus and Kefauver, 2018). These technologies have shown the potential to reduce labor requirements in the assessment of “breeder-preferred” traits and, in some cases, can deliver more detailed information about the biophysical crop parameters. Usually, most efforts in this field are focused on using low-cost RGB (visible), multispectral/hyperspectral, light detection and ranging (LIDAR) or thermal infrared imaging sensors. Detailed information can be found in the literature about different applications for field phenotyping using these sensors (Araus and Cairns, 2014; Deery et al., 2014; Araus et al., 2018). For example, applications of digital RGB sensors in FBP include visible imaging to estimate leaf color, crop ear counting, canopy cover, or canopy height (Kefauver et al., 2015; Holman et al., 2016; Fernandez-Gallego et al., 2019). Spectral imaging sensors are normally used to derive the spectral response of the vegetation and their biophysical traits such as leaf water content, chlorophyll and xanthophyll levels, biomass or the leaf area index (LAI) (Li et al., 2014; Mazis et al., 2020). Thermal imaging has been used to estimate plant water status (Romano et al., 2011; Prashar and Jones, 2014), and LIDAR point clouds to estimate vegetation structural parameters (Madec et al., 2017; Jimenez-Berni et al., 2018). However, most of the breeding programs focused on these targets have tended to use RS technologies to phenotype annual crops. Such studies are rarely performed in woody crops. To the best of our knowledge, only Virlet et al. (2014); Ampatzidis et al. (2019); Coupel-Ledru et al. (2019); Gutiérrez-Gordillo et al. (2020), and López-Granados et al. (2019) have used RS imagery for FBP in woody crops such as apple, citrus and almond.

As previously mentioned, there is an urgent need to identify rootstocks with improved WUE, which, for instance, could be planted in drylands or to cope with scarce water supplies. For this purpose, it is critical to develop tools capable of determining actual transpiration rates at canopy level which can be widely used in breeding programs. Until now, the field phenotyping response of woody crops to water use constraints has constituted a bottleneck for breeding programs due to the complexity of measuring actual transpiration or water status in a large number of trees (Virlet et al., 2014). The few studies that have been published were conducted using high-throughput phenotyping platforms deployed in greenhouses and under controlled conditions, which have the advantage that plants in pots can be weighed and biomass estimated from imagery (Pereyra-Irujo et al., 2012; Lopez et al., 2015).

In recent years, improvements in computational performance, open-source programming languages, lower data requirements, and the simplification of different complex approaches used to estimate actual crop evapotranspiration (ET_*a*_) through RS have contributed, at least in part, to reducing the existing gap between RS physical modeling methods and agricultural applications. Among the different methods, the surface energy balance (SEB) models are probably the most complex to run, but at the same time provide high accuracy and robustness in estimating ET_*a*_ in different environments (Norman et al., 1995; Bastiaanssen et al., 1998; Mecikalski et al., 1999; Allen et al., 2007; Boulet et al., 2015). These models have mostly been used for assessing the spatial and temporal variability of ET_*a*_ at regional and field scale using satellite imagery (Semmens et al., 2016; He et al., 2017; Knipper et al., 2019), although some of them have also been used with very high-resolution aircraft imagery (Hoffman et al., 2016; Xia et al., 2016; Nieto et al., 2019). Among the different SEB models, the two-source energy balance (TSEB) modeling scheme allows the possibility to estimate transpiration and evaporation separately (Norman et al., 1995), by using the Priestley-Taylor approach (Priestley and Taylor, 1972) when radiometric temperature (*T*_*rad*_) is obtained from satellite imagery (e.g., Knipper et al., 2019), or through a contextual approach if high-resolution thermal imagery is available, in which case it is possible to directly obtain soil (*T*_*s*_) and canopy (*T*_*c*_) surface temperatures (Nieto et al., 2019). The TSEB is a two-source model built on the Shuttleworth-Wallace (S-W) energy combination model which can be used to estimate potential evapotranspiration (ET_*p*_) and its partition components separately (Shuttleworth and Wallace, 1985).

Based on the hypothesis that the ratio between ET_*a*_ and ET_*p*_ can be used as a crop water stress indicator, this paper aims to demonstrate the potential of the TSEB and S-W models for phenotyping and breeding purposes in woody crops. Differences in the amount of evapotranspired water and water status will be explored in the almond cultivar “Marinada” grafted onto a collection of 10 rootstocks irrigated under different water regimes. Different RS approaches to determine certain biophysical traits of the vegetation are also explored and the values obtained are used as inputs of the TSEB and S-W models.

## Materials and Methods

### Study Site and Experimental Design

**FIGURE 1:**
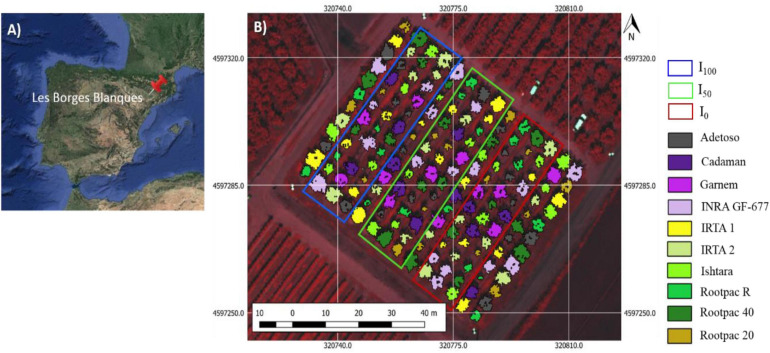
Location of the field experiment, observing in **(A)** the study site located at the IRTA experimental station in Les Borges Blanques (Lleida, Spain), and **(B)** design of the almond rootstock trial with three irrigation treatments (I_100_, I_50_, and I_0_).

The study was carried out in an experimental almond orchard located at the experimental station of IRTA (Institute of Research and Technology, Food and Agriculture) in Les Borges Blanques, Spain (41°30′31.89″N; 0°51′10.70″E, 323 m elevation) during the 2018 and 2019 growing seasons (Figure 1). The climate in the area is Mediterranean, with annual rainfall of 535 and 377 mm for 2018 and 2019, respectively. The orchard is the result of a rootstock trial planted in 2010 which used cv. “Marinada” as the scion cultivar (Vargas et al., 2008) and the following rootstocks: Adesoto, Cadaman^®^, Garnem^®^, INRA GF-677, IRTA 1, IRTA 2, Ishtara^®^, Rootpac^®^ R, Rootpac^®^ 40, and Rootpac^®^ 20 (Table 1). Trees were planted at a spacing distance of 4.5 m with 5.0 m between rows, and trained to an open vase system.

**TABLE 1:** List of evaluated rootstock, parentage, origin and tested cultivar.

**Rootstock**	**Parentage**	**Origin**
Adesoto	Clonal selection of *Prunus insititia*	CSIC-Aula Dei (Spain)
Cadaman^®^	*Prunus persica* × *Prunus davidiana*	IFGO (Hungary) and INRA
Garnem^®^	*Prunus dulcis* × *Prunus persica*	CITA (Spain)
INRA GF-677	*Prunus dulcis* × *Prunus persica*	INRA (France)
IRTA-1	*Prunus dulcis* × *Prunus persica*	IRTA (Spain)
IRTA-2	*Prunus cerasifera* × *Prunus dulcis*	IRTA (Spain)
Ishtara^®^	*(Prunus cerasifera* × *Prunus salicina)* × *(Prunus cerasifera* × *Prunus persica)*	INRA (France)
Rootpac^®^ R	*Prunus cerasifera* × *Prunus dulcis*	Agromillora Iberia (Spain)
Rootpac^®^ 40	(*Prunus dulcis* × *Prunus persica*) × (*Prunus dulcis* × *Prunus persica*	Agromillora Iberia (Spain)
Rootpac^®^ 20	*Prunus besseyi* × *Prunus cerasifera*	Agromillora Iberia (Spain)

The study followed a split-plot design, where irrigation treatment is the main plot and the rootstocks are the sub-plots. The trial consisted of three irrigation treatments: (i) conventional irrigation (I_100_), receiving 100% of ET_*c*_ during the whole irrigation season; (ii) half irrigation (I_50_), receiving 50% of ET_*c*_ during the whole irrigation season, and (iii) deficit irrigation (I_0_), which received 100% of ET_*c*_ during the whole irrigation season except for ∼30 days before the airborne campaign when irrigation was halted. The total amount of water applied in I_100_ throughout the growing season (from April to October) was 652 mm and 618 mm in 2018 and 2019, respectively. Each treatment had three repetitions, each in a row, with the 10 different rootstocks in each row. Rootstock distribution within each row followed a randomized design. One additional row was included between treatments for protection.

Trees were irrigated on a daily basis calculating water requirements through a water balance method for replacing crop evapotranspiration (ET_*c*_) as follows: ET_*c*_ = (ET_*o*_ x Kc)–effective rainfall. The ET_*o*_ was collected from the public network of weather stations closest to the study site (Xarxa Agrometeorològica de Catalunya (XAC), and Servei Meterorològic de Catalunya., 2020), which uses the Penman-Monteith method (Allen et al., 1998) to calculate it. Annual ET_*o*_ was 1061 and 1133 mm in 2018 and 2019, respectively. The Kc used were derived from Goldhamer (2012): Kc_1_ = 0.70 (April), Kc_2_ = 0.95 (May), Kc_3_ = 1.09 (June), Kc_4_ = 1.15 (July), Kc_5_ = 1.17 (August), and Kc_6_ = 1.12 (September). Effective rainfall was estimated as half of the rainfall for a single event-day with more than 10 mm of precipitation; otherwise was considered to be zero. The irrigation system consisted of two drip lines, with fifteen drippers per tree (3.5 L h^–1^ per dripper). Soil texture was clay-loam and the effective soil depth was ∼150 cm. Tree management for pruning, diseases and pests control, soil management and fertilization was based on Spanish integrated production management practices (BOE, 2002).

### Image Collection

**FIGURE 2:**
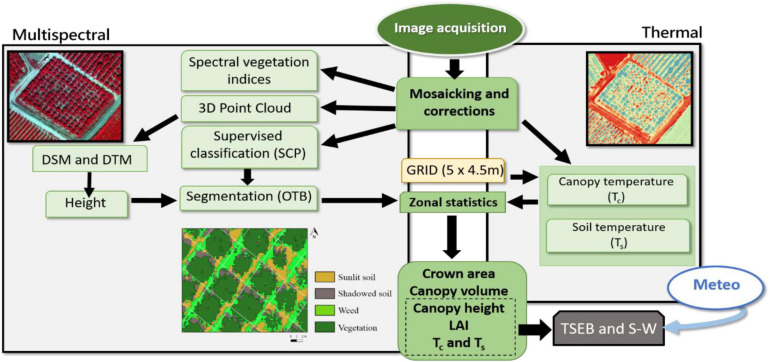
Flowchart of the procedures used for processing the multispectral and thermal images in order to obtain the different biophysical variables of the vegetation and some of the inputs for the two-source energy balance (TSEB) and Shuttleworth and Wallace (S-W) models.

The airborne campaign was conducted on 24th July and 28th of August 2018, and on 24th July 2019. Air temperature (T_*a*_) and vapor pressure deficit (VPD) at the moment of image acquisition were, respectively, 33.4°C and 2.9 kPa for 24th July 2018, 31.3°C and 2.2 kPa for 28th August 2018, and 34.4°C and 3.6 kPa for 24th July 2019. Flights were conducted at 12:00 solar time (14:00 local time) with a thermal (FLIR SC655, FLIR Systems, Wilsonville, OR, United States) and multispectral sensor (MACAW, Tetracam, Chatsworth, CA, United States) on board a manned aircraft. Flight altitude was ∼200 m above ground level, providing thermal and multispectral images at ∼0.25 and 0.03 m pixel^–1^ average resolution, respectively. The thermal sensor has a spectral response in the range of 7.5–13 μm and an image resolution of 640 × 480 pixels. The optical focal length is 13.1 mm, yielding an angular field of view of 45°. The sensor has a focal plane array based on uncooled microbolometers. The MACAW sensor has 1.4 mega-pixel complementary metal-oxide semiconductor (CMOS) sensors with a 9.6 mm fixed lens. These provide images of 1,280 × 1,024 pixels. The sensor contains six user-selectable narrow band filters at 10 nm full width at half maximum (FWHM), with center wavelengths at 515.3, 570.9, 682.2, 710.5, 781.1, and 871.8 nm. The thermal sensor was connected to a laptop via ethernet, and the multispectral camera via USB 3.0 protocol. All thermal and multispectral images were radiometrically, atmospherically and geometrically corrected. The radiometric calibration of the thermal sensor was assessed in the laboratory using a blackbody (model P80P, Land Instruments, Dronfield, United Kingdom). The radiometric calibration of the multispectral sensor was conducted through an external incident light sensor which measured the irradiance levels of light at the same bands as the MACAW multispectral sensor. In addition, *in situ* spectral measurements in ground calibration targets were performed using a Jaz spectrometer (Ocean Optics, Inc., Dunedin, FL, United States). The Jaz has a wavelength response from 200 to 1,100 nm and an optical resolution of ∼0.3–10.0 nm. During spectral collection, spectrometer calibration measurements were taken with a reference panel (white color Spectralon) and dark current before and after taking readings from radiometric calibration targets. In addition, a range of field calibrations were conducted through *in situ* surface temperature measurements in ground calibration targets using a portable IR gun (Fluke 62 mini IR thermometer, Everett, WA, United States). Geometric correction was conducted using ground control points (GCP), and measuring the position in each with a handheld GPS (Global Positioning System) (Geo7×, Trimble GeoExplorer series, Sunnyvale, CA, United States) with a precision of ∼0.20 cm. All images were mosaicked using the Agisoft Metashape Professional software (Agisoft LLC., St. Petersburg, Russia) and geometrically and radiometrically terrain corrected with QGIS 3.4 (QGIS 3.4.15). Figure 2 shows the flowchart of the procedures used to process the images and obtain the information of the different parameters.

### Field Measurements

The fraction of photosynthetically active radiation (PAR) intercepted by the canopy (*fiPAR*) was measured on the same clear days as image acquisition from 11:00 to 14:00 h (local time) using a portable linear ceptometer (AccuPAR model LP-80, Decagon Devices Inc., Pullman, WA, United States). Incident PAR above and below the canopy was measured for each tree. Twenty PAR readings were recorded below each tree canopy covering the tree spacing. The ceptometer was placed in a horizontal position at ground level perpendicular to the row. The *fiPAR* was calculated by dividing the averaged PAR below the canopy by the incident PAR taken in full sunlight at an open site with no interference from the canopy. The LAI was derived by means of *fiPAR*, using the Norman-Jarvis model (Norman and Jarvis, 1974) and assuming a leaf absorptivity for light at 0.9. Daily *fiPAR* (*fiPAR*_*d*_) was estimated using an hourly model of light interception (Oyarzun et al., 2007). In the model, the porosity parameter was estimated so that the simulated hourly intercepted value at noon equalled the instantaneous value measured in the field. Then, *fiPAR*_*d*_ was calculated by integrating the diurnal course of the simulated *fiPAR*. Tree architectural parameters such as canopy height, crown width perpendicular to and along rows, and branch insertion height were also measured.

Concomitant to image acquisition, one midday Ψ_*stem*_ was measured in each tree. Shaded leaves were selected and kept in a plastic bag covered by aluminum foil for 2 h before the measurement in order to equilibrate the water potential between leaf, stem and branches. All measurements were acquired in less than 2 h with a pressure chamber (Plant Water Status Console, Model 3500; Soil Moisture Equipment Corp., Santa Barbara, CA) and following the protocol established by Shackel et al. (1997).

### Biophysical Traits of the Vegetation

Three different approaches were tested to estimate LAI and *fiPAR*_*d*_: (i) estimates of canopy height and volume through photogrammetry, (ii) spectral vegetation indices (VIs), and (iii) the PROSAIL radiative transfer model.

The three-dimensional (3D) tree canopy volume was obtained following the protocol described by Caruso et al. (2019). The digital surface model (DSM) was generated from the photogrammetric point cloud of multispectral images. A classification of bare ground pixels located between tree rows were used to obtain the digital terrain model (DTM) of the orchard. Then, a raster corresponding to heights (from the ground to maximum height of the canopy) was obtained by subtracting the DTM from the DSM using the raster calculator tool of the QGIS software.

The semi-automatic OS v.6 classification plugin of the QGIS software (Congelo, 2016) was used to classify vegetation, sunlit and shadowed bare soil and weeds (Figure 2). Then, the vegetation mask was used to delineate each crown area through the watershed object-based segmentation algorithm included in the Orfeo Toolbox, and to obtain the average height and crown area of each individual tree. Canopy volume of each pixel was obtained by multiplying the pixel area by its corresponding height value (from the ground to the maximum height within the pixel) (Caruso et al., 2019). The total volume of each tree was obtained by adding all the canopy pixels. Finally, the net canopy volume was calculated by subtracting the volume comprised between the ground and the branch insertion of the canopy from the total volume of each tree.

Several spectral VIs were obtained from multispectral images (Table 2). These indices have been shown to be closely related to certain specific features of plant structure and have demonstrated a great potential to estimate the LAI (Haboudane et al., 2004). Besides the extensively used normalized difference vegetation index (NDVI), this study tested different indices within the red-edge spectral region. The red-edge region is characterized by a sharp change in vegetation reflectance due to chlorophyll absorption, and it has been demonstrated that this is strongly influenced by the LAI (Delegido et al., 2013; Xie et al., 2018).

**TABLE 2:** List of spectral vegetation indices (VI), their formulation and reference.

**Index**	**Formula**	**References**
NDVI	(*R*_870_−*R*_680_)/(*R*_870_ + *R*_680_)	Rouse et al., 1973
GNDVI	(*R*_870_−*R*_570_)/(*R*_870_ + *R*_570_)	Gitelson et al., 1996
MCARI	[(*R*_710_−*R*_6__8__0_)−0.2 (*R*_71__0_-*R*_57__0_)] *R*_71__0_/*R*_6__8__0_	Daughtry et al., 2000
NDRE	(*R*_870_−*R*_710_)/(*R*_870_ + *R*_710_)	Barnes et al., 2000
MSRRE	*(R*_870_*/ R*_710_)−1/ $\sqrt{R_{870}+R_{710}+1}$	Wu et al., 2008

**R is defined as reflectance.**

The LAI and *fiPAR* were also estimated following the protocol described by Weiss and Baret (2016), which retrieves these parameters from Sentinel-2 bands. Instead, this study used the six very-high resolution spectral bands of the multispectral sensor. The method consists of generating a large comprehensive dataset of vegetation characteristics, covering all possible ranges in the vegetation parameters described in Table 3, after which simulated reflectance factors are obtained by running the PROSAIL model (Jacquemoud et al., 2009) in forward mode. With these two arrays of values (vegetation parameters and simulated spectra), a neural network was built per each parameter (many-to-one relation). Finally, the trained neural network was applied to the multispectral images for each tree, computing the average reflectance of a rectangular grid with tree spacing distance (4.5 × 5.0 m), in order to predict the biophysical parameters from the reflectances acquired by the multispectral camera.

**TABLE 3:** List of parameters and their ranges used in PROSAIL reflectance modeling.

**Image acquisition**	**24th July 2018**	**28th August 2018**	**24th July 2019**
DOY	205	240	205
Time image acquisition	12.50	12.25	12.25
Solar irradiance (W.m^–2^)	924	778	910
Solar zenith angle (°)	21.81	32.04	21.38
Solar azimuth angle (°)	193.43	184.53	183.91
Spectral bands (nm)	515.3, 570.9, 682.2, 710.5, 781.1, 871.8		
Soil reflectance	0.121, 0.163, 0.192, 0.319, 0.373, 0.363		
Number of simulations	100,000		
Latitude	41.5		
Longitude	0.85		
N_*leaf*_	1.2–2.2		
C_*ab*_ (μg.cm^–2^)	0–90		
C_*ar*_ (μg.cm^–2^)	0–40		
C_*brown*_	0.0–1.0		
C_*w*_ (g.cm^–2^)	0.003–0.011		
C_*d*__*m*_ (g.cm^–2^)	0.003–0.011		
LAI	0.0–6.0		
Average leaf angle (°)	30–80		
Hotspot (m.m^–1^)	0.1–0.5		

**N_*leaf*_, Leaf mesophyll structure parameter; C_*ab*_, Leaf chlorophyll content; C_*ar*_, Carotenoids content; C_*brown*_, Leaf brown pigments content; C_*w*_, Leaf water content; C_*d*__*m*_, Leaf dry matter content.**

### Evapotranspiration and Crop Water Stress Index

The TSEB model was used to estimate ET_*a*_ and its partition between soil and vegetation. One of the main advantages of TSEB is that it estimates evaporation (E) and transpiration (T) separately using information from T_*rad*_ and biophysical parameters of the vegetation, which are available from RS. The TSEB was originally formulated by Norman et al. (1995) and further improved by Kustas and Anderson (2009). The energy balance is based on the principle of conservation of energy, which calculates latent heat flux as a residual of the surface energy equation (Eq. 1):

(1a) LE≈R-nH-G

(1b) LE≈SR,n-SH-SG

(1c) LE≈CR,n-CHC

where LE is the latent heat flux (W m^–2^), R_*n*_ is the net radiation flux (W m^–2^), G is the soil heat flux (W m^–2^), and H is the sensible heat flux (W m^–2^). The subscripts *c* and *s* refer to canopy and soil, respectively. Surface soil heat flux around solar noon (G) is often calculated in TSEB as a constant fraction of R_*n*_,_*S*_.

Sensible heat flux (H) is partitioned into soil (H_*s*_) and canopy (H_*c*_) fluxes, in which heat flux transport between soil and canopy are connected in series following an analogy of Ohm’s law for electric transport:

(2a) $$H_{s}=\rho \,C_{p}\,\frac{T_{s}-T_{a\,c}}{r_{s}}$$

(2b) $$H_{c}=\rho \,C_{p}\,\frac{T_{c}-T_{a\,c}}{r_{x}}$$

(2c) $$H_{s}+H_{c}=H=\rho \,C_{p}\,\frac{T_{a\,c}-T_{a}}{r_{a\,h}}$$

where ρ is the air density (kg m^–3^), *C*_*p*_ is the specific heat of air (J kg K^–1^), *T*_*s*_ is the soil temperature (K), *T*_*a*_ is the air temperature (K), *T*_*ac*_ is the air temperature in the canopy layer (K), *r*_*s*_ is the resistance to heat flow in the boundary layer immediately above the soil surface (s m^–1^), *r*_*x*_ is the total boundary layer resistance of the complete canopy leaves (s m^–1^), and *r*_*ah*_ is the aerodynamic resistance (s m^–1^) to turbulent heat transport between the air-canopy layer and the overlying air layer.

When TSEB runs with coarse resolution satellite-derived images, soil and canopy temperature cannot be directly retrieved. In such cases, T_*c*_ and T_*s*_ are estimated in an iterative process in which it is first assumed that green canopy transpires at a potential rate based on the Priesley-Taylor equation (Priestley and Taylor, 1972). In this study, however, the high spatial resolution imagery allowed direct retrieval of T_*s*_ and T_*c*_ without the need to compute an initial canopy transpiration (Nieto et al., 2019). That is, T_*c*_ and T_*s*_ were individually obtained for each tree and for the bare soil pixels within the 5 × 4.5 m square grid, respectively. For this purpose, the previously mentioned supervised classification was used, and T_*s*_ corresponded to the averaged sunlit and shadowed bare soil pixels within each grid.

As in other TSEB models, this methodology also requires LAI to calculate radiation partitioning as well as wind attenuation through the canopy toward the soil surface. Ground measurements of LAI were used in the TSEB. Ancillary variables that were needed, such as meteorological data, were obtained from the closest weather station to the study site (XAC, Les Borges Blanques: 41°30′40.85″N; 0°51′22.21″E). Given T_*c*_ and T_*s*_, the heat fluxes from the soil and canopy can be derived directly using Eqs. (2a,b) and the sensible heat flux from Eq. (2c). Actual evapotranspiration at the instant of aircraft image acquisition (ET_*inst*_) was calculated as:

(3) $$E\,T_{i\,n\,s\,t}=3600\,\frac{L\,E}{I\,\rho_{w}}$$

where ET_*inst*_ is the instantaneous ET (mm h^–1^), ρ_*w*_ represents the density of water (1,000 kg m^–3^), and λ is the latent heat of vaporization (J kg^–1^). Then, ET_*inst*_ was upscaled to daily water fluxes, in units of mm/day, by multiplying the instantaneous ratio between latent heat flux and solar irradiance by average daily solar irradiance (Cammalleri et al., 2014).

The ET_*p*_ was retrieved from the S-W model (Shuttleworth and Wallace, 1985). This model also considers two coupled sources in a resistance network: the transpiration from vegetation and the evaporation from substrate soil. The theoretical basis of the S-W model is the Penman-Monteith energy combination equation, and includes two parts, one for the soil surface and the other for the plant surface. The potential evapotranspiration and transpiration computed by the S-W model, setting a minimum stomatal resistance value of 100 sm^–1^, are then used as the basis for estimating the theoretical metrics of the crop water stress index (CWSI). In this study, the CWSI was calculated as:

(4) $$C\,W\,S\,I=1-\frac{E\,T_{a}}{E\,T_{p}}$$

where ET_*a*_ and ET_*p*_ correspond to actual and potential evapotranspiration, estimated, respectively, from the TSEB and S-W models.

### Statistical Analysis

Data was analyzed using the JMP^®^ statistical software (SAS Institute Inc., SAS Campus Drive, Cary, NC, United States). Estimates of LAI and *fiPAR*_*d*_ were also derived using a stepwise multiple regression analysis which included the VIs and canopy volume estimates as dependent variables. All the variables were evaluated with a three-way analysis of variance (three-way ANOVA). Statistical significance was established for *P* < 0.05. Tukey’s HSD test was applied to separate least square means that differed significantly.

## Results

### Estimates of the Biophysical Variables of the Vegetation

**FIGURE 3:**
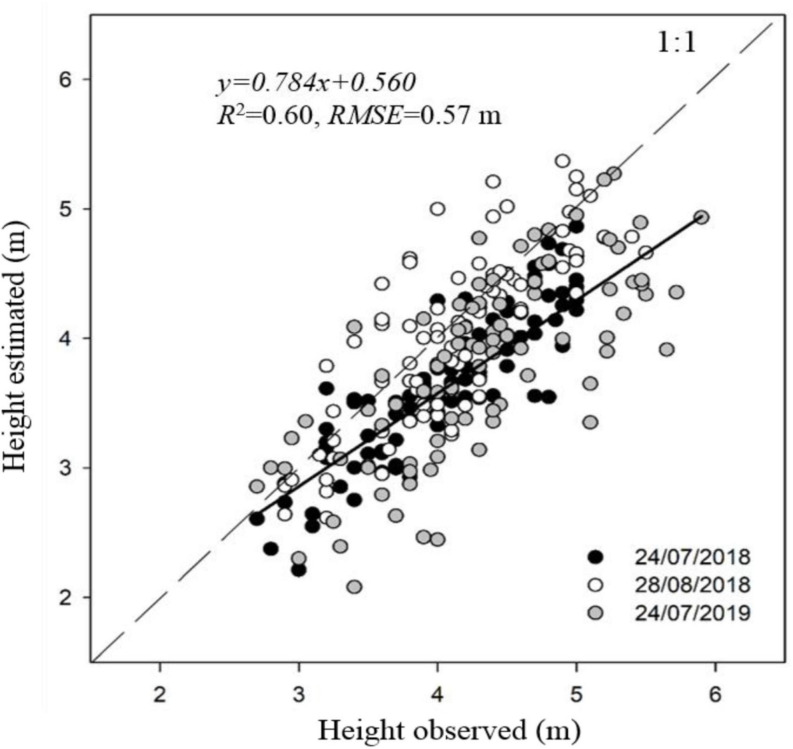
Comparison between ground measured and airborne-estimated maximum canopy height of almond trees on 24th July and 28th August 2018 and 24th July 2019. Linear regression corresponds to aggregated data of the three dates.

The one-to-one relationship between observed and estimated canopy height was significant for the three dates of image acquisition, with *R*^2^-values ranging from 0.54 to 0.77 and RMSE values from 0.43 to 0.65 m. The *R*^2^ and RMSE were, respectively, 0.60 and 0.57 m when aggregating data from the three dates (Figure 3). Values of measured canopy height and LAI ranged between 2.7–5.9 m and 0.3–2.0 m m^–1^, respectively. Estimates of crown area and canopy volume through photogrammetry were linearly related with *fiPAR*_*d*_ and LAI, with *R*^2^ ranging from 0.38 to 0.72 (Table 4), and being slightly higher for LAI. Non-significant differences were found when estimating these parameters either through crown area or canopy volume, in part because canopy height (used to estimate canopy volume) was quadratically correlated with crown area (*R*^2^ = 0.60, *p* < 0.001). All the tested spectral VIs were significant and linearly correlated with LAI and *fiPAR*_*d*_ when the data was analyzed for individual dates, but most of the regressions were not significant when the data from the three dates was aggregated. The modified chlorophyll absorption in reflectance index (MCARI) showed the lowest *R*^2^ in all cases. The NDVI and normalized difference red-edge (NDRE) index had the highest *R*^2^ with LAI on 28th August 2018 and 24th July 2019. In addition, NDRE had the highest *R*^2^ on 24th July 2018. On that day, estimates of LAI through NDVI, MCARI and the green normalized difference vegetation index (GNDVI) showed the lowest *R*^2^. The VIs with the highest *R*^2^ with *fiPAR*_*d*_ were similar to those reported for LAI. The use of the radiative transfer model PROSAIL significantly improved the estimates of LAI and *fiPAR*_*d*_ in comparison to the use of simple VIs. The *R*^2^ and RMSE for LAI ranged from 0.46 to 0.67 and from 0.24 to 0.39 m m^–1^, respectively, and for *fiPAR*_*d*_ from 0.45 to 0.64 and from 0.07 to 0.14%, respectively. In addition, when the data from the three dates were analyzed together, the *R*^2^ and RMSE were, respectively 0.40 and 0.34 m m^–1^ for LAI and 0.29 and 0.12% for *fiPAR*_*d*_.

**TABLE 4:** Coefficients of determination (*R*^2^) of the regressions between leaf area index (LAI) and daily fraction of intercepted radiation (*fiPAR*_*d*_) with spectral vegetation indices (VIs), crown area and canopy volume, PROSAIL radiative transfer model, and multiple regression analysis with empirical variables.

**Parameters**	**NDVI**	**GNDVI**	**MCARI**	**NDRE**	**MSRre**	**Crown area (m^2^)**	**Canopy volume (m^3^)**	**Predicted LAI and *fiPAR* (PROSAIL)**	**Multiple regression analysis**
LAI_24/7/__2018_	0.24	0.25	0.30	0.56	0.54	0.72	0.72	y = 0.45x+0.60, *R*^2^ = 0.67, RMSE = 0.24	y = −0.74+3.31NDRE+0.03Volume, *R*^2^ = 0.74, RMSE = 0.19
LAI_28/8/__2018_	0.57	0.50	0.49	0.51	0.48	0.65	0.64	y = 0.57x+0.20, *R*^2^ = 0.46, RMSE = 0.38	y = −1.81+6.93GNDVI-1.98MSRre+ 0.02Volume, *R*^2^ = 0.70, RMSE = 0.17
LAI_24/7/__2019_	0.41	0.41	0.36	0.42	0.41	0.44	0.49	y = 1.00x+0.05, *R*^2^ = 0.56, RMSE = 0.39	y = −1.22+3.50NDRE+ 0.02Volume, *R*^2^ = 0.54, RMSE = 0.30
LAI_*all*_	ns	ns	ns	0.15	ns	0.59	0.58	y = 0.59x+0.49, *R*^2^ = 0.40, RMSE = 0.34	y = 0.49+1.98NDRE-1.06NDVI+0.03Volume, *R*^2^ = 0.60, RMSE = 0.24
*fiPAR*_*d* 24/7/__2018_	0.37	0.39	0.15	0.49	0.46	0.53	0.50	y = 0.54x+0.28, *R*^2^ = 0.64, RMSE = 0.07	y = −0.91+0.05MSRre+ 2.22NDVI+0.01Volume, *R*^2^ = 0.64, RMSE = 0.06
*fiPAR*_*d* 28/8/__2018_	0.45	0.49	0.38	0.47	0.46	0.49	0.45	y = 0.80x+0.09, *R*^2^ = 0.45, RMSE = 0.14	y = 0.03+0.83GNDVI+0.01Volume, *R*^2^ = 0.56, RMSE = 0.05
*fiPAR*_*d* 24/7/__2019_	0.38	0.40	0.32	0.41	0.39	0.38	0.43	y = 1.15x−0.15, *R*^2^ = 0.51, RMSE = 0.14	y = −2.93–3.21MSRre+12.75NDRE, *R*^2^ = 0.53, RMSE = 0.10
*fiPAR*_*d all*_	ns	0.18	ns	0.16	ns	0.49	0.48	y = 0.44x+0.34, *R*^2^ = 0.29, RMSE = 0.12	y = −0.24+0.62GNDVI+6.95MCARI+ 1.19NDRE-0.65NDVI+0.01Volume, *R*^2^ = 0.56, RMSE = 0.07
									

**FIGURE 4:**
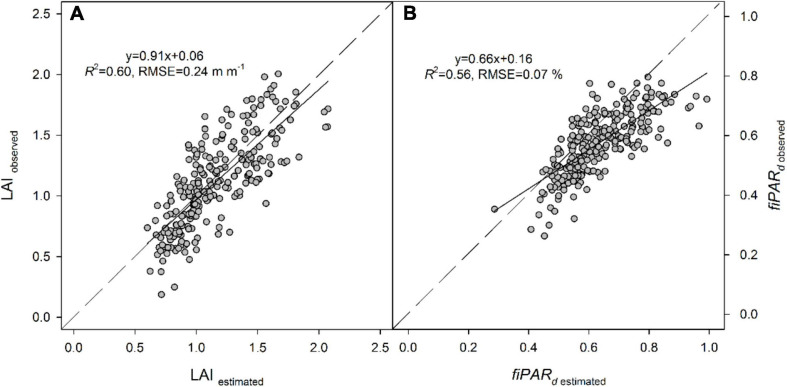
Relationships between observed and estimated **(A)** LAI and **(B)** *fiPAR*_*d*_ in almond trees, calculated from the equations obtained in the multiple regression analysis for the three dates together (LAI = 0.49+1.98NDRE-1.06NDVI+0.03Volume; *fiPAR*_*d*_ = –0.24+0.62GNDVI+6.95MCARI+1.19NDRE-0.65NDVI+0.01Volume).

The multiple regression analysis using the empirical variables slightly increased the predictions of LAI and *fiPAR*_*d*_ in all cases. Results indicated that the best predictions were obtained when canopy volume was combined with other VIs, which varied between dates. Overall, the best predictions of LAI and *fiPAR*_*d*_ using the three dates of data together were observed with the multiple regression analysis. The *R*^2^ and RMSE were, respectively, 0.60 and 0.22 m m^–1^ for LAI and 0.56 and 0.07% for *fiPAR*_*d*_ (Table 4 and Figure 4).

### Comparison Between Rootstocks

The analysis of variance showed that the rootstock source was significant for all the evaluated variables (*p* < 0.0001) and that the *treatment x rootstock* interactions were not significant, except for Ψ_*stem*_ (Table 5). Significant differences between treatments and for the *date x treatment* interaction were also observed for Ψ_*stem*_ (*p* < 0.0001). The remotely sensed estimates of crown area, canopy volume, LAI and *fiPAR*_*d*_ were significant for the interaction *date x rootstock*. The date source was also significant for ET_*a*_, ET_*a*_/*fiPAR_*d*_*, and kernel yield.

**TABLE 5:** Results of an analysis of variance (three-way ANOVA) testing the factor effects (date, treatment and rootstock) on the different variables estimated through remote sensing.

**Variables/Source**	**Area**	**Volume**	**LAI**	***fiPAR*_*d*_**	**Ψ_*stem*_**	**T_*c*_-T_*a*_**	**ET_*a*_**	**ET_*a*_/*fiPAR*_*d*_**	**CWSI**	**Kernel yield**
Date	ns	ns	ns	ns	ns	ns	<.0001*	<.0001*	ns	<.0001*
Treatment	ns	ns	Ns	ns	<.0001*	ns	Ns	ns	ns	ns
Rootstock	<.0001*	<.0001*	<.0001*	<.0001*	<.0001*	<.0001*	<.0001*	<.0001*	<.0001*	<.0001*
Date*Rootstock	0.0242*	0.0001*	<.0001*	0.0076*	ns	ns	ns	ns	ns	<.0001*
Date*Treatment	ns	ns	Ns	ns	0.0084*	ns	ns	ns	ns	ns
Rootstock*Treatment	ns	ns	Ns	ns	0.033*	ns	ns	ns	ns	ns
Date*Rootstock*Treatment	ns	ns	Ns	ns	ns	ns	ns	ns	ns	ns

*Corresponds to significant differences at p ≤ 0.05; ns, not significant.

**FIGURE 5:**
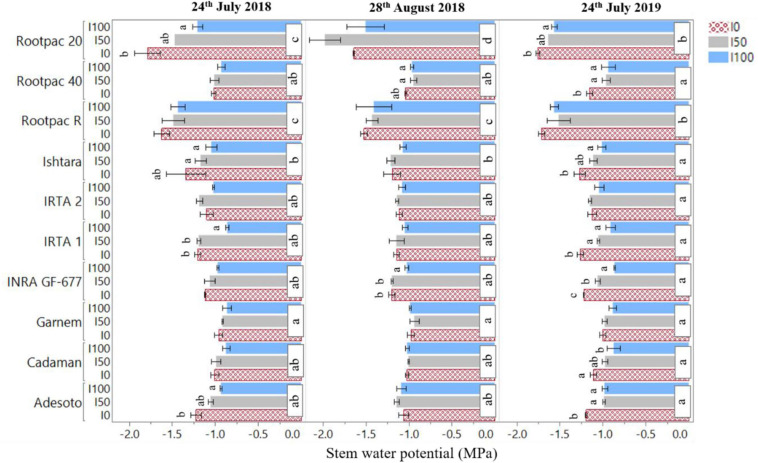
Differences in stem water potential (Ψ_*stem*_) between rootstock and irrigation treatments (I_100_, I_50_, I_0_) for the three dates of image acquisition (24th July and 28th August 2018 and 24th July 2019). Letters indicate statistically significant differences between rootstock (*P* < 0.05, Tukey’s HSD test).

Overall, Cadaman^®^ and Garnem^®^ had the highest crown area, canopy volume, LAI and *fiPAR*_*d*_, followed by INRA GF-677 (Table 6). On the other hand, Rootpac^®^ 20 had the lowest values for all the evaluated variables. Non-significant differences were detected between IRTA 1, IRTA 2, Ishtara^®^, Rootpac^®^ R, Rootpac^®^ 40, and Adesoto. Figure 5 shows the significant differences in Ψ_*stem*_ between rootstock and irrigation treatments. The results show that Rootpac^®^ R and Rootpac^®^ 20 were the two rootstocks with the lowest Ψ_*stem*_ for the three measured dates. However, the latter had slightly lower values, mostly during 2018. On the other hand, Garnem^®^, Cadaman^®^, Adesoto, INRA GF-677, IRTA 1, IRTA 2, and Rootpac^®^ 40 displayed similar behavior for the three dates, showing the highest Ψ_*stem*_ values. Measurements conducted on 24th July 2018 showed significant differences between treatments in Adesoto, IRTA 1, Ishtara^®^ and Rootpac^®^ 20. Significant differences in Ψ_*stem*_ for 28th August 2018 were only observed in INRA GF-677 and Rootpac^®^ 40. On 24th July 2019, all rootstocks except Garnem^®^, IRTA 2 and Rootpac^®^ R had significant differences in Ψ_*stem*_ between irrigation treatments. In all cases, the I_0_ treatment tended to have the lowest Ψ_*stem*_ values.

**TABLE 6:** Comparison of crown area, canopy volume, leaf area index (LAI) and daily fraction of intercepted radiation (*fiPAR*_*d*_) between almond rootstocks for each image acquisition date.

**Date**	**Rootstock/Variable**	**Adesoto**	**Cadaman^®^**	**Garnem^®^**	**INRA GF-677**	**IRTA 1**	**IRTA 2**	**Ishtara^®^**	**Rootpac^®^ R**	**Rootpac^®^ 40**	**Rootpac^®^ 20**
24th July 2018	3.29 ef	10.97 a	11.30 a	8.72 b	5.31 cde	6.02 cd	6.09 cd	4.36 cde	6.97 bc	2.58 f
28th August 2018		8.34 cde	14.12 a	11.03 abc	9.51 bcd	9.02 bcd	7.21 de	7.09 de	8.62 cd	4.98 e	5.18 e
24th July 2019		4.58 fg	12.15 ab	12.62 a	10.02 bc	6.37 def	7.24 de	6.98 def	5.48 efg	8.21 cd	2.96 g
Mean		6.52 c	12.41 a	11.65 a	9.41 b	6.90 c	6.82 c	6.74 c	6.15 c	6.79 c	3.74 d
24th July 2018	4.81 ef	23.58 a	26.29 a	17.58 b	8.46 cde	10.87 cd	10.81 cd	6.77 def	12.81 c	3.04 f
28th August 2018		20.98 bc	36.32 a	28.52 ab	18.85 bc	19.55 bc	13.20 cd	14.24 cd	19.77 bc	7.39 d	8.11 d
24th July 2019		6.87 fg	29.86 ab	33.08 a	23.66 bc	11.87 ef	16.65 de	12.24 ef	8.46 fg	19.31 cd	2.87 g
Mean		10.57 c	29.92 a	29.21 a	20.03 b	13.29 c	13.57 c	12.49 c	11.67 c	13.39 c	5.07 d
24th July 2018	0.66 d	1.46 a	1.51 a	1.33 ab	0.84 cd	0.93 cd	0.96 cd	0.73 d	1.09 bc	0.60 d
28th August 2018		1.22 bcd	1.57 a	1.34 abc	1.27 abcd	1.19 bcd	0.98 def	1.05 cde	1.17 bcd	0.81 f	0.90 ef
24th July 2019		0.79 ef	1.25 abcd	1.69 a	1.60 ab	1.08 cde	1.08 cde	1.32 abc	0.81 def	1.23 bcd	0.46 f
Mean		0.92 bc	1.44 a	1.51 a	1.39 a	1.04 b	0.99 b	1.08 b	0.91 bc	1.05 b	0.67 c
24th July 2018	0.47	0.63 ab	0.65 a	0.60 abc	0.50 cde	0.51 bcd	0.54 abcd	0.46 de	0.55 abcd	0.41 e
28th August 2018		0.68 ab	0.70 a	0.67 abc	0.64 abcd	0.61 abcd	0.57 cde	0.59 bcde	0.61 bcd	0.51 e	0.54 de
24th July 2019		0.49 de	0.61 abcd	0.68 ab	0.69 a	0.56 cd	0.56 bcd	0.65 abc	0.53 d	0.60 abcd	0.39 e
Mean		0.54 b	0.65 a	0.66 a	0.64 a	0.55 b	0.55 b	0.59 ab	0.53 b	0.55 b	0.45 c

**Different letters mean significant differences between rootstocks at p ≤ 0.05 using Tukey’s honest significant difference test.**

**FIGURE 6:**
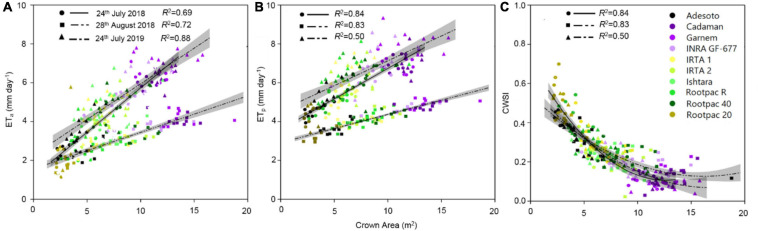
Relationships between estimated canopy crown area and **(A)** actual evapotranspiration (ET_*a*_), **(B)** potential evapotranspiration (ET_*p*_) and **(C)** CWSI, calculated as 1-ET_*a*_/ET_0_, for the three dates of image acquisition (24th July 2018 and 2019 and 28th August 2018). Shadowed lines indicate the 95% confidence intervals of the regression models.

**FIGURE 7:**
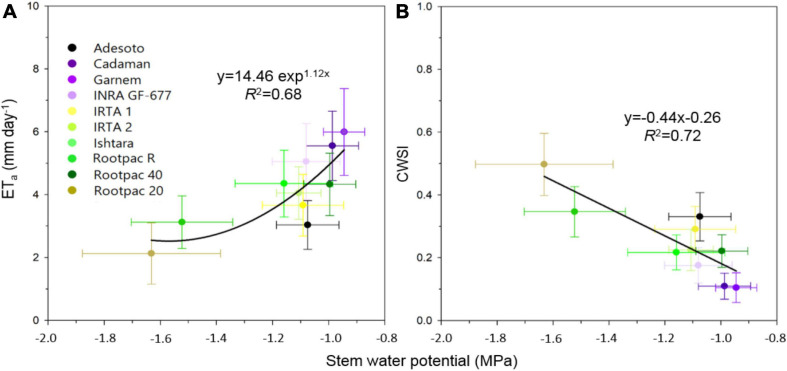
Relationships between stem water potential (Ψ_*stem*_) and **(A)** actual evapotranspiration (ET_*a*_), and **(B)** crop water stress index (CWSI) calculated as 1-ET_*a*_/ET_*p*_.

Among other parameters, LAI and T_*c*_ are inputs required by the TSEB model to estimate the ET_*a*_ of a crop. In this study, differences in canopy to air temperature (T_*c*_-T_*a*_) between rootstocks were also significant and agreed with Ψ_*stem*_ measurements. More specifically, the relationships between T_*c*_-T_*a*_ and Ψ_*stem*_ had *R*^2^ values of 0.57, 0.60, and 0.53 for 24th July 2018, 28th August 2018 and 24th July 2019, respectively (graphs not shown). The relationships between ET_*a*_ with T_*c*_-T_*a*_ and LAI gave respective *R*^2^-values of 0.57 and 0.87 for 24th July 2018, 0.66 and 0.87 for 28th August 2018, and 0.63 and 0.68 for 24th July 2019 (graphs not shown). These results suggest that ET_*a*_ had a stronger relationship with LAI than with T_*c*_-T_*a*_, probably due to the lack of range in T_*c*_-T_*a*_ values. In fact, both ET_*a*_ and ET_*p*_ were also positive and linearly correlated with the canopy crown area (Figures 6A,B). Values of ET_*a*_ ranged from 1.8 to 8 mm day^–1^, depending on date and rootstock. For a given crown area, ET_*a*_ values varied between dates, with ET_*a*_ rates corresponding to 28th August 2018 lower than those of 24th July 2018 and 24th July 2019 (Figure 6A). These differences in ET_*a*_ between dates were more pronounced as crown area increased. The highest ET_*a*_ and ET_*p*_ were observed in Cadaman^®^ and Garnem^®^ in the three dates, followed by INRA GF-677. On the other hand, Rootpac^®^ 20 was the rootstock with the lowest ET_*a*_ and ET_*p*_. Adesoto and Rootpac^®^ R also had low ET_*a*_ and ET_*p*_ values. When differences between dates were atmospherically normalized through the CWSI, all the data followed the same polynomial regression, indicating that rootstocks with a low crown area (Rootpac^®^ 20) also seemed to be more stressed than those with higher crown areas (Cadaman^®^ and Garnem^®^) (Figure 6C). Maximum CWSI values reached ∼0.6 for trees with a crown area of ∼2.5 m^–2^. The relationship between averaged ET_*a*_ and Ψ_*stem*_ was significant (Figure 7A), as was the regression between CWSI and Ψ_*stem*_ (Figure 7B). These regressions indicate that trees grafted on the least vigorous rootstocks (Rootpac^®^ 20 and Rootpac^®^ R) were also those with the lowest Ψ_*stem*_ values. Accordingly, these two rootstocks also had the highest CWSI and lowest ET_*a*_ rates, with values ranging from 1.4 to 5.3 mm day^–1^. Of these two rootstocks, Rootpac^®^ 20 had the lowest Ψ_*stem*_ and ET_*a*_.

**FIGURE 8:**
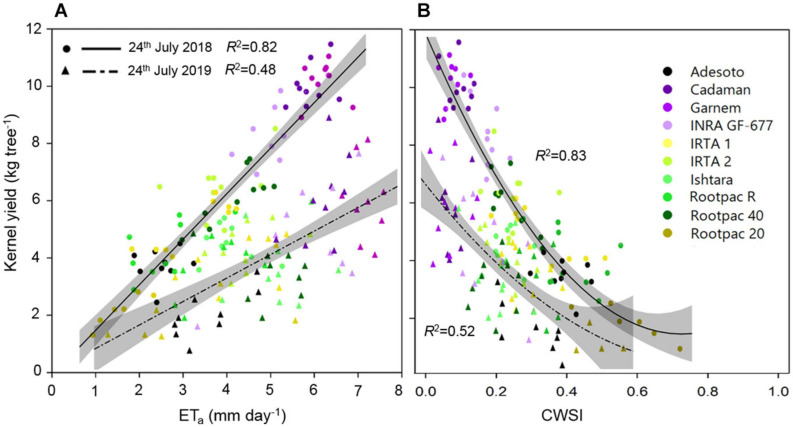
Relationships between kernel yield (kg tree^–1^) and **(A)** actual evapotranspiration (ET_*a*_) and **(B)** CWSI of the different rootstocks estimated on 24th July 2018 and 2019. Shadowed lines indicate the 95% confidence intervals of the regression models.

It can be seen in Figure 8A that kernel yield was positively linearly related to ET_*a*_ in both years, although the *R*^2^ varied between them. It can also be seen that kernel yield tended to decrease as CWSI increased, reaching minimum yields at CWSI values of around 0.5–0.7 (Figure 8B).

## Discussion

The effect of rootstock on tree canopy vigor has been widely reported through *in situ* measurements of TSCA, canopy volume or LAI (Russo et al., 2007; Gullo et al., 2014; Mestre et al., 2015; Yahmed et al., 2016; Lordan et al., 2019). However, this study demonstrates the feasibility of using very high-resolution multispectral airborne imagery to estimate the architectural traits of the vegetation in an almond rootstock trial and to use them to estimate ET_*a*_.

The results confirm that the best fit to estimate LAI and *fiPAR*_*d*_ was through the combination of information derived from photogrammetry and VIs (Table 4). The highest *R*^2^ values with both LAI and *fiPAR*_*d*_ were obtained when photogrammetric techniques were used to estimate crown area and canopy volume. Since the latter depends on canopy height, which showed an RMSE of 0.57 m (Figure 3), it is possible that any advance in accuracy when estimating canopy height could also contribute to improving estimates of LAI and *fiPAR*_*d*_. Increasing the number of images acquired from different viewing angles, higher overlap, or lower flying altitude in order to describe the full 3D scene and avoid occlusion effects are some of the ways that could help to improve canopy height estimates. Other authors have been able to estimate canopy height with greater accuracy. For instance, Zarco-Tejada et al. (2014) and Caruso et al. (2019) obtained RMSE values of 0.22 and 0.35 m, respectively, in olive trees. However, the difference between these two studies and ours was flight altitude (∼130 m of difference) and the trajectories taken by the unmanned aerial vehicle platform which ensured larger image overlaps and point cloud densities.

The use of the PROSAIL model did not improve the estimates of LAI and *fiPAR*_*d*_ in comparison to the multiple regression analysis, probably because this model was not designed for sparse canopies with multiple layers, as is the case of almond orchards (Berger et al., 2018). Until now, PROSAIL has been mostly used to estimate LAI and *fiPAR* with multispectral satellite imagery in non-woody vegetation canopies such as croplands (Duan et al., 2014; Li et al., 2015) and grasslands (Darvishzadeh et al., 2008; Casas et al., 2014), as PROSAIL assumes a homogeneous canopy of randomly placed leaves. However, the model has barely been used in woody crops in combination with very high resolution airborne multispectral imagery. This study also showed that PROSAIL tends to overestimate LAI (Table 4).

On the other hand, it is well-established that VIs are strongly influenced by canopy architecture, optical properties, sun illumination angle, viewing properties and soil background (Huete, 1988; Guillen-Climent et al., 2012; Xie et al., 2018; Prudnikova et al., 2019). In addition, saturations at moderate-to-dense canopies, leaf area distribution, and clumping effect are three of the most important issues influencing the accuracy of optical LAI estimates in row crops (Delalieux et al., 2008; Shafian et al., 2018; Yan et al., 2019). For instance, our study showed that NDVI, GNDVI and MCARI had low *R*^2^ with LAI and *fiPAR*_*d*_ on 24th July 2018, probably caused by a soil background effect. The previous week, and up to 3 days before the flight, a series of rainfall events occurred at the study site amounting to a total precipitation value of 20.2 mm. These events resulted in the moist soil (i.e., “darker”) absorbing more light than other days, mostly in the visible and NIR bands, and therefore affecting the values provided by the indices that used these bands. On the other hand, since most of these parameters are taken into consideration in the PROSAIL model, estimates of LAI and *fiPAR*_*d*_ tended to be better and more consistent over time, although with a systematic overestimation. The methodology used in this study to obtain the biophysical variables of the vegetation was the same as that developed for the Sentinel-2 toolbox (Weiss and Baret, 2016). In that case, a database containing the input radiative transfer model variables was generated first. Then, the corresponding top-of-canopy reflectance for the eight Sentinel-2 bands were simulated with the PROSAIL model. In contrast, in our study we used the six bands derived from the MACAW multispectral sensor. It is also possible that the use of different and a lower number of bands slightly affected the estimates of the biophysical variables.

In this study, all the estimates of the structural parameters of the vegetation indicated that the most dwarfing rootstock was Rootpac^®^ 20, followed by Rootpac^®^ R, Rootpac^®^ 40, Adesoto, Ishtara, IRTA 1, and IRTA 2. Garnem^®^, Cadaman^®^, and INRA GF-677 provided the highest values for the same structural traits. These results are in agreement with, for instance, those reported by Lordan et al. (2019), who evaluated tree canopy vigor in the same rootstock trial for a longer period and also identified Garnem^®^, Cadaman^®^ and INRA GF-677 as those with the greatest tree volume, and Rootpac^®^ 20 as the most dwarfing rootstock in the trial. In agreement, Yahmed et al. (2016) also observed that Garnem^®^ and Rootpac^®^ 40 were, respectively the most and medium vigorous rootstocks and that scions grafted on Rootpac^®^ 20 were the most dwarfing.

The observed differences in ET_*a*_ between dates could be attributable to changes in atmospheric water demand, plant response (stomatal closure) due to water stress, or some phenological effect. In this case study, water stress can be discarded because Ψ_*stem*_ values of the date with the lowest ET_*a*_ (28th August 2018) were slightly less negative in comparison to the other two dates, and because the same behavior was observed with the estimates of ET_*p*_ with the S-W model (Figure 6B). Our hypothesis for the lower ET_*a*_ values observed for 28th August 2018 is that these are associated with a lower atmospheric demand of water, since the midday VPD and daily solar irradiance (R_*s*_) for that day were slightly lower (VPD = 2.2 KPa and R_*s*_ = 195 W m^–2^) than the other 2 days (respectively, 2.9 KPa and 319 W m^–2^ for 24th July 2018 and 3.6 KPa and 294 W m^–2^ for 24th July 2019). Accordingly, T_*c*_-T_*a*_ values for that day were also higher. Several studies have published non-water-stressed baselines (NWSB)for different crops, which consist in relating T_*c*_-T_*a*_ with VPD at midday for well-watered trees (Bellvert et al., 2016; García-Tejero et al., 2018; Gonzalez-Dugo et al., 2019; Gutiérrez-Gordillo et al., 2020). These regressions indicate that T_*c*_-T_*a*_ tended to decrease as VPD increased. In addition, Bellvert et al. (2018) showed that the regression between T_*c*_-T_*a*_ and VPD in California almonds was sensitive to the phenology, indicating that for a given increase in VPD, early growth stages, which correspond to vegetative growth (shell expansion and hardening), have more transpiration cooling than the kernel and post-kernel filling stages.

Although the amount of water applied in the different irrigation treatments was the same for all rootstocks, the response of most of the evaluated parameters varied between rootstocks, particularly for Ψ_*stem*_ where the *rootstock x treatment* and *date x treatment* interactions were significant (Table 5). As seen in Figure 7, the least vigorous rootstocks (Rootpac^®^ 20, Rootpac^®^ R) had the lowest Ψ_*stem*_ and ET_*a*_ values. However, Rootpac^®^ 20 had slightly lowest Ψ_*stem*_ than Rootpac^®^ R. These rootstocks are characterized by having *Prunus cerasifera* (myrobolan) as one of the parents, which may lead to a slight and delayed “localized” incompatibility between plum-almond species, as has previously been described in cherry and peach/plum (Treutter and Feucht, 1991) or almond/plum (Bernhard and Grasselly, 1959) combinations. This type of incompatibility is characterized by anatomical irregularities at the rootstock/scion union interface with breaks in vascular connections, which, in turn, prevent quick resumption of the growth of both root and canopy (Errea et al., 2001; Leonardi and Romano, 2004). It has also been demonstrated that trees grafted on dwarfing rootstocks such as Rootpac^®^ 20 and Rootpac^®^ R tend to have lower Ψ_*stem*_ values, and that this is likely related to the lower water absorption capability of the root system to satisfy the transpiration demand of the canopy (Yahmed et al., 2016). In our case, defoliation and yellowing problems were also observed in some trees of the I_0_ treatment. The lower Ψ_*stem*_ observed in Rootpac^®^ 20 could be explained because this rootstock was obtained by crossing two plum species (*Prunus besseyi*× *Prunus cerasifera*), and therefore probably displaying a smaller root system, while Rootpac^®^ R had a higher compatibility with the scion because at least has a *Prunus dulcis* as one of the parents.

In terms of WUE or drought tolerance, several studies have related canopy vigor and root system with the level of tolerance (Serra et al., 2014; Zhang et al., 2016). The hypothesis is that vigorous plants are usually more tolerant due to a bigger root system, and vice versa. However, a comparison between rootstocks with statistical differences in canopy vigor is not always the most appropriate method because both plant water demand and the amount of water available in the soil per unit of canopy vigor will differ depending on canopy size and may therefore lead to inappropriate interpretations of the results. In this study, in order to explain the differences between rootstocks, we grouped them according to canopy vigor (mean of canopy volume) (Table 7), and then analyzed the statistical differences in the relations between Ψ_*stem*_ and ET_*a*_ within each group by using data of the three flights. A first group, which contained Garnem^®^, Cadaman^®^ and INRA GF-677, was characterized by having the highest ET_*a*_ rates due to high canopy volume and probably a longer root system which permitted a higher water absorption capacity. Concurring with this finding, Black et al. (2010) described Cadaman^®^ as a rootstock with a high root biomass. The ANCOVA analysis showed no significant differences between rootstocks in the ET_*a*_ vs. Ψ_*stem*_ regressions of the group 1 (*p* = 0.721) (Table 8). Despite of this, it seems that INRA GF-677 had slightly lower Ψ_*stem*_ and ET_*a*_ values and a higher CWSI. A second group with medium canopy vigor rootstocks was composed of Rootpac^®^ 40, Adesoto, IRTA 1, IRTA 2, Ishtara^®^, and Rootpac^®^ R. Rootpac^®^ R had by some way the lowest Ψ_*stem*_ values, which together with Adesoto and IRTA 1 corresponded with the lowest ET_*a*_ rates, without significant differences among them. However, the low Ψ_*stem*_ of Rootpac^®^ R suggests that this rootstock was acting as if it had a lower hydraulic conductivity or root biomass in comparison to the others which caused a fall in Ψ_*stem*_. The ANCOVA analysis of group 2 only showed significant differences between rootstock in the intercept (*p* = 0.034) (Table 8). Therefore, as there were not significant differences between slopes, we cannot affirm that these rootstocks have differences in the root hydraulic resistance (*R*_*root*_). In order to improve our understanding of the response of rootstocks to water stress, future studies should be able to determine the hydraulic resistances of different rootstocks through measurements of water potential gradients and transpiration (López-Bernal et al., 2015). Differences in the intercept could be explained either due to still small differences in the canopy volume between rootstocks of group 2 or due to a physiological response related with an anisohydric or isohydric behavior. In fact, the rootstock with the significantly lower intercept (Adesoto) was the one with the lowest crown area (Figure 6). The last group consisted solely of Rootpac^®^ 20, which had the lowest Ψ_*stem*_ and ET_*a*_ values. Opazo et al. (2020) compared Rootpac^®^ 20 and Rootpac^®^ 40 and reported that plants grafted on the former had lower transpiration rates, less root biomass and proved to be less tolerant to drought than the latter. Results obtained in our study reinforce these observations (Table 7).

**TABLE 7:** Mean of the variables Ψ_*stem*_, ET_*a*_, and CWSI, and slope and intercept of the regression ET_*a*_ vs. Ψ_*stem*_ for each rootstock grouped on the basis of the analysis of variance of canopy volume.

**Rootstock**	**Group by canopy volume**	**Ψ_*stem*_**	**ET_*a*_**	**CWSI**	**Slope**	**Intercept**
Garnem^®^	1	−0.95 ± 0.07a	5.99*a*	0.10*b*	10.13	15.46
Cadaman^®^	1	−0.99 ± 0.07a	5.55*ab*	0.11*b*	5.59	11.07
INRA GF-677	1	−1.08 ± 0.12 b	5.05*b*	0.18*a*	2.66	7.92
Rootpac^®^ 40	2	−0.99 ± 0.09 a	4.32*a*	0.22*c*	–0.01	4.43
Adetoso	2	−1.07 ± 0.11 ab	3.07*b*	0.33*ab*	3.91	7.12
IRTA 1	2	−1.09 ± 0.15 ab	3.66*ab*	0.29*b*	2.99	6.93
IRTA 2	2	−1.11 ± 0.07 b	4.04*a*	0.22*c*	0.34	4.35
Ishtara^®^	2	−1.16 ± 0.17 b	4.35*a*	0.22*c*	0.84	5.33
Rootpac^®^ R	2	−1.52 ± 0.18 c	3.12*b*−	0.34*a*	0.67	4.29
Rootpac^®^ 20	3	−1.63 ± 0.24 −	2.12−	0.49−	0.66	3.14

Different letters mean significant differences within each group at p ≤ 0.05 using Tukey’s honest significant difference test. – means that no statistical analysis was performed. *Corresponds to significant differences at p ≤ 0.05

**TABLE 8:** Analysis of covariance (ANCOVA) of the relationships between ET_*a*_ and Ψ_*stem*_ shown in Table 7 for rootstocks of groups 1 and 2.

	**Source**	**g.l**	**Sum squares**	**Mean square**	**F**	**Prob > F**	**HSD Tukey**	
Group 1	Model	5	28.71	5.74	4.13	0.003*	Garnem^®^	5.56 a
	Error	68	94.57	1.39			Cadaman^®^	5.37 a
	Total	73	123.28				INRA GF-677	5.22 a
	Ψ_*stem*_	1	15.99		11.49	0.001*		
	Rootstock	2	0.91		0.32	0.721		
	Rootstock * Ψ_*stem*_	2	3.84		1.38	0.257		
Group 2	Model	11	42.52	3.86	5.46	<.0001*	Rootpac^®^ 40	3.99 ab
	Error	129	91.39	0.71			Adesoto	2.87 c
	Total	140	133.92				IRTA 1	3.49 bc
	Ψ_*stem*_	1	3.26		4.61	0.034*	IRTA 2	3.97 ab
	Rootstock	5	21.73		6.13	<.0001*	Ishtara^®^	4.22 a
	Rootstock * Ψ_*stem*_	5	3.18		0.89	0.495	Rootpac^®^ R	3.62 bc

*Different letters mean significant differences between rootstocks at p ≤ 0.05 using Tukey’s honest significant difference test.*

The establishment of the relationship between crop yield and the consumptive use of water (the so-called production function) in row crops is of particular interest, but at the same time is not easy to obtain due to the need for long-term studies and the difficulty in assessing consumptive use (Goldhamer and Fereres, 2017). Although many studies have demonstrated that almonds are one of the species able to maintain high kernel yield under deficit irrigation conditions (Torrecillas et al., 1989; Girona et al., 2005; Egea et al., 2010), other studies have reported that yield is dependent on canopy PAR light interception, and therefore this will increase with *fiPAR*_*d*_ (Jin et al., 2020). In our study, the rootstocks with the highest canopy volumes and *fiPAR*_*d*_ (Cadaman^®^ and Garnem^®^) had the highest ET_*a*_ and yields, while the lowest yields were observed in those which had the lowest ET_*a*_ (Rootpac^®^ 20, followed by Rootpac^®^ 40 and Rootpac^®^ R) (Figure 8A). It should also be noted that the *R*^2^ of both the yield-ET_*a*_ and yield-CWSI regressions were higher in 2018 than in 2019, because the former had higher yield while the latter coincided with an alternate bearing year.

**FIGURE 9:**
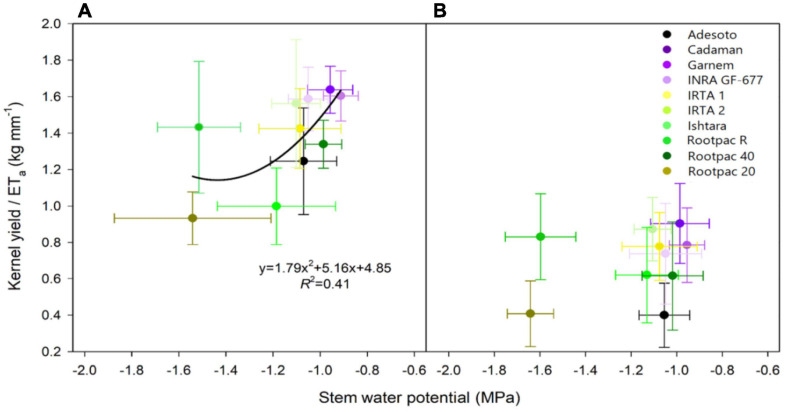
Relationships between kernel yield/ET_*a*_ (kg tree^–1^ / mm of water evapotranspired) and stem water potential (Ψ_*stem*_) for **(A)** 24th July 2018, and **(B)** 24th July 2019.

This study also shows the daily water production function as yield per unit of water evapotranspired, using data from 24th July 2018 to 24th July 2019. Figure 9 shows that water productivity (kernel yield/mm water evapotranspired) differed between rootstocks and that the regression with Ψ_*stem*_ tended to decrease as water stress increased. This regression was significant for 2018 (Figure 9A) but not for 2019 (Figure 9B). The rootstocks in the previously mentioned first group (Garnem^®^ and Cadaman^®^) showed the highest water productivity in both years, together with INRA GF-677, IRTA 1, IRTA 2, and Rootpac^®^ 40. Although Adesoto and Ishtara^®^ had similar high Ψ_*stem*_ values, water productivity was slightly lower. Interestingly, despite the negative Ψ_*stem*_ of Rootpac^®^ R, water productivity values were similar to those obtained in the rootstocks in group 1. This is attributable to the significantly higher yield of Rootpac^®^ R, despite having Ψ_*stem*_ and ET_*a*_ values similar to Rootpac^®^ 20.

## Conclusion

This study has demonstrated, for the first time, the feasibility of using a surface energy balance model for high-throughput phenotyping of crop evapotranspiration in an almond rootstock collection. The analysis allowed the quantification of the following almond traits that are of paramount importance in rootstock phenotyping: canopy tree height, crown area, canopy volume, LAI, *fiPAR*_*d*_, actual and potential crop evapotranspiration, and the crop water stress index. The LAI and *fiPAR*_*d*_ were, respectively, estimated with an *R*^2^ of 0.60 and 0.56 through a multiple linear regression equation, which included estimates of both parameters obtained from spectral vegetation indices and estimates of crown area and canopy volume through photogrammetry techniques. Cadaman^®^ and Garnem^®^ were identified as the rootstocks with the highest canopy vigor as well as the highest ET_*a*_. These two rootstocks were characterized by maintaining high Ψ_*stem*_ values despite reducing the amount of irrigation water applied. In contrast, Rootpac^®^ 20 and Rootpac^®^ R had the lowest canopy vigor and ET_*a*_, and also the lowest Ψ_*stem*_ in the I_100_ treatment suggesting that this was due to a localized incompatibility between plum-almond species, differences in the root system and/or low hydraulic conductivity. Other rootstocks had medium canopy vigor. Of these, Adesoto and IRTA 1 had the lowest ET_*a*_ values and Rootpac^®^ 40 and Ishtara the highest. Yield was linearly related with ET_*a*_. Cadaman^®^ and Garnem^®^ also had the highest water productivity, and Rootpac^®^ 20 and Rootpac^®^ R the lowest. However, the water productivity of Rootpac^®^ R was significantly higher than that of Rootpac^®^ 20.

The use of energy balance models such as the TSEB using very high-resolution imagery opens the possibility to efficiently evaluate the WUE of a crop in many other different rootstock collections or varieties located in different environments. This will improve the manner in which field phenotyping has been applied until now and will help crop breeders to better understand and identify the rootstocks/varieties best adapted to drought. In addition, since the TSEB allows the partitioning of plant transpiration and surface evaporation components, future studies will focus on using transpiration instead of ET_*a*_, and together with measurements of water potential gradients, to determine differences in root hydraulic resistances.

## Data Availability Statement

Publicly available datasets were analyzed in this study. This data can be found here: https://github.com/hectornieto.

## Author Contributions

JB wrote the manuscript and analyzed the remote sensing and field data. HN was the developer of the pyTSEB code used in this study. AP processed all the images and did the preliminary analysis. CJ-Č conducted the radiometric calibration of images and the analysis with the PROSAIL model. LZ conducted field measurements during the airborne campaign. XM designed the experimental design, collaborated in the field measurements campaign and provided critical insights into the manuscript writing. All authors contributed to the article and approved the submitted version.

## Conflict of Interest

The authors declare that the research was conducted in the absence of any commercial or financial relationships that could be construed as a potential conflict of interest.
